# Molecular Mechanisms for the Regulation of Insulin-Stimulated Glucose Uptake by Small Guanosine Triphosphatases in Skeletal Muscle and Adipocytes

**DOI:** 10.3390/ijms151018677

**Published:** 2014-10-16

**Authors:** Takaya Satoh

**Affiliations:** Laboratory of Cell Biology, Department of Biological Science, Graduate School of Science, Osaka Prefecture University, 1-1 Gakuen-cho, Naka-ku, Sakai, Osaka 599-8531, Japan; E-Mail: tkysato@b.s.osakafu-u.ac.jp; Tel./Fax: +81-72-254-7650.

**Keywords:** adipocyte, glucose uptake, GLUT4, insulin, skeletal muscle, small GTPase

## Abstract

Insulin is a hormone that regulates the blood glucose level by stimulating various physiological responses in its target tissues. In skeletal muscle and adipose tissue, insulin promotes membrane trafficking of the glucose transporter GLUT4 from GLUT4 storage vesicles to the plasma membrane, thereby facilitating the uptake of glucose from the circulation. Detailed mechanisms underlying insulin-dependent intracellular signal transduction for glucose uptake remain largely unknown. In this article, I give an overview on the recently identified signaling network involving Rab, Ras, and Rho family small guanosine triphosphatases (GTPases) that regulates glucose uptake in insulin-responsive tissues. In particular, the regulatory mechanisms for these small GTPases and the cross-talk between protein kinase and small GTPase cascades are highlighted.

## 1. Insulin-Dependent Glucose Uptake Mediated by Subcellular Redistribution of the Glucose Transporter

Insulin is an anabolic hormone that acts on various target tissues, including the liver, skeletal muscle, and fat tissue, regulating the blood glucose level [1,2,3,4]. The activity of enzymes that govern metabolic responses, such as glycogen synthesis, glycogenolysis, gluconeogenesis, and lipogenesis, is rigorously controlled via intracellular signaling mechanisms downstream of the insulin receptor. Additionally, insulin promotes the uptake of circulating glucose into its target tissues, such as skeletal muscle and fat tissue, and thereby reduces the blood glucose level. In healthy individuals, a large fraction of glucose disposal stimulated by insulin occurs in skeletal muscle, and glucose disposal in fat tissue is relatively small. Obesity causes the loss of sensitivity to insulin in target tissues, a phenomenon known as insulin resistance.

Glucose uptake in response to insulin in skeletal muscle and adipose tissue is mediated by the glucose transporter GLUT4 [1,2,3,4]. GLUT4 is a 12-transmembrane protein that permits peripheral blood glucose to move into the cell across the plasma membrane. GLUT4-mediated glucose transport down the concentration gradient does not require ATP, and is classified as facilitated diffusion.

**Figure 1:**
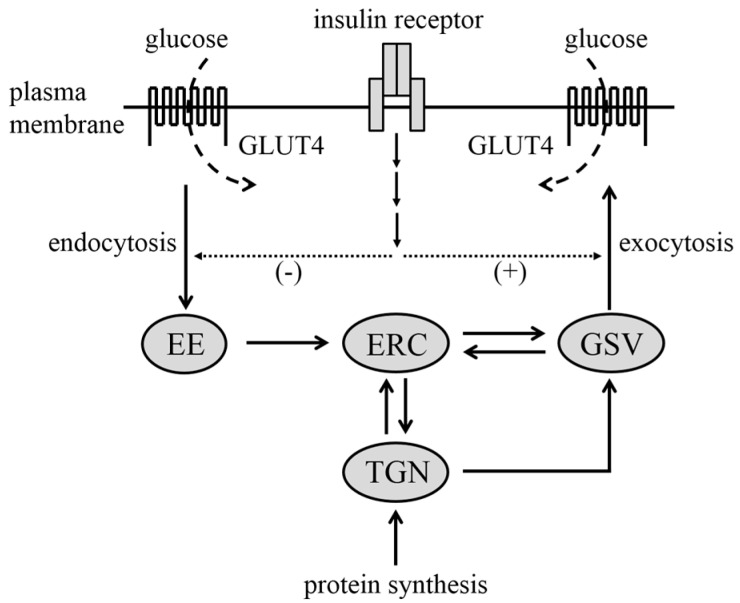
Insulin regulation of intracellular trafficking of GLUT4. Intracellular compartments in which GLUT4 is localized are shown as gray ellipses. Translocation of GLUT4 between compartments is shown by thick solid arrows. Insulin enhances exocytosis and suppresses endocytosis of GLUT4, leading to the redistribution of GLUT4 from GSVs to the plasma membrane as shown by thin dotted arrows. Insulin also regulates other steps of vesicular trafficking (not shown). EE, early endosome; ERC, endosomal recycling compartment; GSV, GLUT4 storage vesicle; TGN, trans-Golgi network.

In unstimulated cells, GLUT4 is sequestered into a specialized intracellular compartment termed GLUT4 storage vesicles (GSVs) (Figure 1) [1,2,3,4]. GLUT4 is also localized in various intracellular membrane compartments, including early endosomes (EEs), the endosomal recycling compartment (ERC), and the trans-Golgi network (TGN). Plasma membrane-localized GLUT4 is incorporated into ERC through endocytosis, and *de novo* synthesized GLUT4 is transported into membrane structures via TGN. Furthermore, these GLUT4 molecules dynamically cycle among intracellular compartments. Following insulin stimulation, GLUT4 is translocated from GSVs to the plasma membrane via an exocytic pathway. Endocytosis of cell surface GLUT4 is also negatively regulated by insulin in adipocytes. In addition, insulin controls multiple steps between intracellular membrane compartments. Consequently, the net accumulation of GLUT4 in the plasma membrane is achieved in response to insulin, enabling transport of circulating glucose into the cell. A critical role of GLUT4-mediated glucose uptake in glucose homeostasis has been demonstrated by studies using transgenic [5,6,7] and gene knockout [8,9] mice. Therefore, it is important to clarify intracellular signaling mechanisms by which insulin stimulates glucose uptake via membrane trafficking of GLUT4-containing vesicles also for clinical purposes.

## 2. Signal Transduction Pathways Downstream of the Insulin Receptor that Lead to GLUT4-Mediated Glucose Uptake

**Figure 2:**
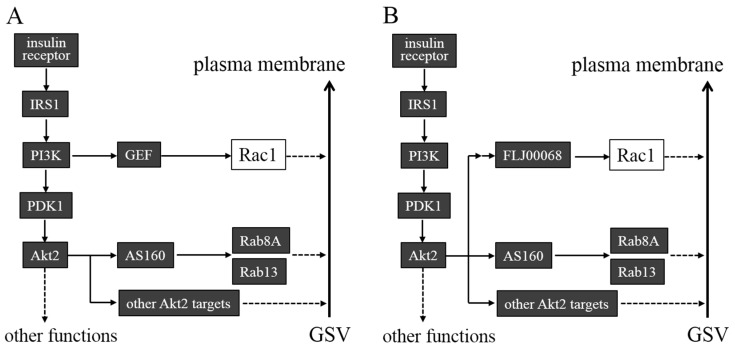
Proposed models for signaling pathways that direct translocation of GLUT4 from GLUT4 storage vesicles (GSVs) to the plasma membrane in skeletal muscle. (**A**) Insulin stimulates a signaling cascade composed of the insulin receptor, the adaptor protein IRS1, phosphoinositide 3-kinase (PI3K), and protein kinases PDK1 and Akt2. Akt2 and the small GTPase Rac1 function independently downstream of PI3K. The guanine nucleotide exchange factor (GEF) that regulates Rac1 is unknown. AS160 is a substrate of Akt2, which acts as a GTPase-activating protein (GAP) for Rab8A and Rab13. Other Akt2 targets may also regulate exocytosis of GLUT4; (**B**) Rac1 is regulated downstream of Akt2. The GEF FLJ00068 has been implicated in the regulation of Rac1 downstream of Akt2.

Insulin exerts its effects on target cells via the activation of the specific receptor (Figure 2). Upon the binding of insulin, protein tyrosine kinase activity of the receptor is enhanced, inducing phosphorylation of diverse target proteins. The adaptor protein IRS1 is a major substrate of the insulin receptor, serving as a platform for the signaling complex. Class I phosphoinositide 3-kinase (PI3K) is a component of this signaling complex, catalyzing phosphorylation in position 3 of the inositol ring of phosphoinositides. Products of PI3K, such as phosphatidylinositol-3,4,5-trisphosphate and phosphatidylinositol-3,4-bisphosphate, bind to pleckstrin homology domains of cAMP-dependent kinase/cGMP-dependent kinase/protein kinase C family protein kinases, such as PDK1 and Akt2, recruiting these kinases to the plasma membrane [10,11]. A specific serine residue of the carboxyl-terminal hydrophobic motif of Akt2 is first phosphorylated, and then translocated PDK1 phosphorylates threonine in the activation segment of Akt2, leading to its full activation.

An array of studies using genetically engineered mice have shown that Akt2 plays a pivotal role in insulin-dependent glucose uptake. Glucose uptake induced by insulin at a low concentration was reduced in soleus and extensor digitorum longus muscles from Akt2-deficient mice compared to wild-type mice [12,13]. Furthermore, Akt2-deficient mice exhibited hyperglycemia, hyperinsulinemia and glucose intolerance [12,13]. Therefore, it is important to reveal the mechanism by which Akt2 directs glucose uptake in response to insulin.

## 3. Role of Rab Proteins and Their Regulators in Akt2-Mediated GLUT4 Translocation in Response to Insulin

Approximately 70 members have been identified in the Rab family of small GTPases in humans. Rab proteins are mostly involved in the regulation of intracellular membrane trafficking, such as vesicle budding, delivery, tethering, and fusion with the target membrane [14,15]. Like other small GTPases, Rab proteins cycle between guanosine 5'-diphosphate (GDP)-bound inactive and guanosine 5'-triphosphate (GTP)-bound active states. Moreover, a cycle of membrane insertion and extraction is coupled with the GDP/GTP cycle in the case of Rab proteins. Three kinds of proteins, guanine nucleotide exchange factor (GEF), GTPase-activating protein (GAP), and GDP dissociation inhibitor (GDI), serve as regulators for the above two cycles of Rab family proteins. GEF enhances the replacement of bound GDP with GTP. GAP stimulates hydrolysis of bound GTP to GDP and inorganic phosphate. Thus, in general, Rab proteins are positively and negatively regulated by GEFs and GAPs, respectively. On the other hand, GDI specifically interacts with the GDP-bound form and masks the lipid moiety at the carboxyl terminus, thereby preserving Rab proteins in the cytosol. When associated with membranes, activated Rab proteins exert their function through specific interactions with their effectors. GAP-stimulated hydrolysis of bound GTP turns off the switch, and the GDP-bound inactive Rab protein moves back to the cytosol through the association with GDI.

Some endosomal Rab proteins, such as Rab4 and Rab5, are localized in GLUT4 vesicles, and have been implicated in the regulation of GLUT4 redistribution in response to insulin [16]. The over-expression of Rab4 indeed affects GLUT4 trafficking [17]. For this action, post-translational modification and GTP loading to Rab4 are important [17]. Rab5 regulates the formation of early endosomes and their transport from the plasma membrane. Inhibition of Rab5 function lowers the internalization of GLUT4 after insulin removal, leading to the increase in the basal level of surface GLUT4 [18].

Rab10 is also present in GLUT4 vesicles, and has been implicated in insulin-stimulated translocation of GLUT4 to the plasma membrane in adipocytes [19,20]. In fact, a constitutively activated Rab10 mutant increased the level of cell surface GLUT4, whereas insulin-stimulated GLUT4 translocation was suppressed by knockdown of Rab10. Recently, Rab10 has been characterized as a marker for GSVs, but not endosomes containing GLUT4 [21]. Using this specific GSV marker, it has been shown that GSVs primarily approach and fuse at the plasma membrane, without interacting with endosomes on their way to the plasma membrane [21]. Other Rab10 subfamily members, Rab8A and Rab13, are expressed in skeletal muscle cells. Rab8A and Rab13 were indeed activated following insulin stimulation, and were also implicated in the regulation of insulin-stimulated GLUT4 translocation [22].

Interestingly, a Tre-2/Bub2/Cdc16 domain-containing Rab-GAP, AS160 (also termed TBC1D4), which specifically acts on Rab8A, Rab10, and Rab13, is a substrate of Akt2 [23]. Considering that GAP negatively regulates the signaling pathway mediated by its target GTPase, AS160 may be an Akt2-directed negative regulator for GLUT4 translocation mediated by Rab10 subfamily members. Actually, the increase in the basal level of plasma membrane-localized GLUT4 due to AS160 knockdown was restored by knocking down Rab10 [19]. Moreover, overexpression of Rab8A or Rab13 suppressed the inhibitory effect of an AS160 mutant having constitutively high GAP activity on basal and insulin-stimulated levels of surface GLUT4 [22].

In unstimulated cells, AS160 is associated with GLUT4 vesicles, and insulin causes dissociation of AS160 from GLUT4 vesicles [24]. Insulin also stimulates phosphorylation of five amino acid residues in AS160 by Akt2 in adipocytes [25]. Phosphorylation of AS160 occurs also in skeletal muscle cells upon insulin stimulation and contraction [26,27]. AS160 phosphorylated by Akt2 shows decreased Rab–GAP activity, leading to the increase in the level of the active GTP-bound form of Rab proteins in GLUT4 vesicles. An AS160 mutant, in which four of the phosphorylation sites are replaced with alanine, but the Rab–GAP catalytic domain is intact, was insensitive to Akt2-dependent negative regulation, and remarkably inhibited insulin-induced GLUT4 translocation in both skeletal muscle and adipocytes [25,28]. Additionally, knockdown of AS160 increased the fraction of cell surface GLUT4 in unstimulated cells [24,29]. Thus, AS160 is thought to be an Akt2 substrate that is critical for insulin-promoted glucose uptake. A close relative of AS160, TBC1D1, is also a substrate of Akt2, and is characterized as a Rab–GAP that regulates insulin-dependent GLUT4 translocation [30].

In many cases, small GTPases are activated in response to upstream signals through the action of their specific GEFs. Therefore, GEFs for Rab GTPases may be possible candidates for activators. Actually, a GEF for Rab10 called Dennd4C is present in GLUT4 vesicles, and has been implicated in insulin-dependent GLUT4 translocation in adipocytes [31]. Currently, the role of Akt2 in insulin-dependent regulation of this GEF remains unclear.

## 4. Other Akt2-Dependent Mechanisms for the Induction of Glucose Uptake in Response to Insulin

Signaling molecules other than AS160 also participate in the regulation of GLUT4 translocation downstream of Akt2. For instance, a C2 domain-containing phosphoprotein termed CDP138 is a substrate of Akt2, and is dynamically associated with GLUT4-containing vesicles and the plasma membrane following insulin stimulation [32]. This protein is in fact required for the regulation of insulin-dependent fusion of GLUT4 vesicles with the plasma membrane in adipocytes [32]. Another example is a protein termed Grp1, a GEF for ARF6 [33]. Grp1 is phosphorylated by Akt2, thereby regulating not only insulin-dependent GLUT4 vesicle formation, but also later steps of GLUT4 recycling [33]. Recently, the small GTPase Rac1-dependent pathway has also been proposed to be regulated by Akt2 in skeletal muscle, although the Akt2 substrate for this regulation remains unidentified (see below).

## 5. Rearrangements of the Cytoskeleton Regulated by Rho Family GTPases in Response to Insulin

**Figure 3:**
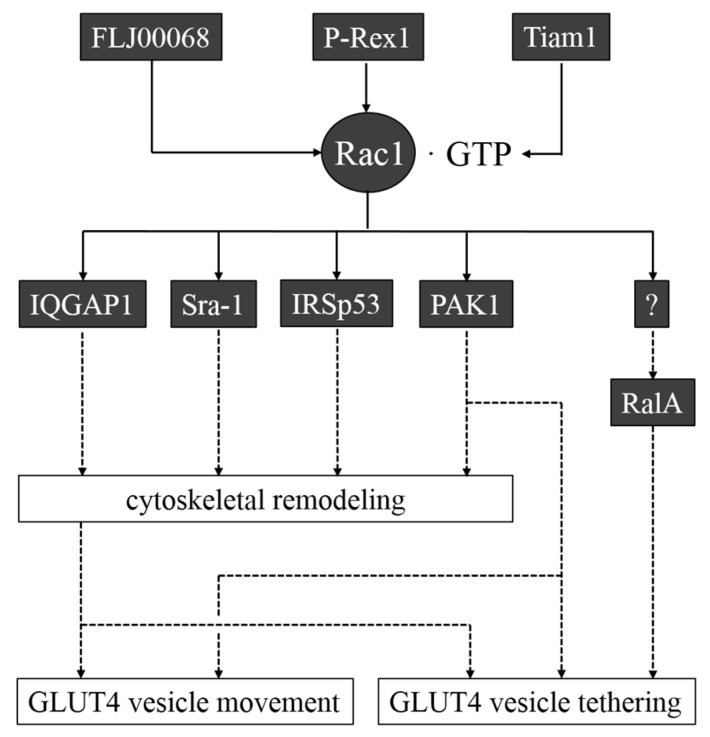
GEFs and target proteins of Rac1 that may be implicated in GLUT4 translocation to the plasma membrane in skeletal muscle and adipocytes. FLJ00068 and P-Rex1 are GEFs that regulate Rac1 activity in skeletal muscle and adipocytes, respectively. Another Rac1 GEF, Tiam1, may also be involved in Rac1 activation. The scaffold protein IQGAP1, the cytoplasmic protein Sra-1, the adaptor protein IRSp53, and the protein kinase PAK1 are Rac1 targets that regulate cytoskeletal remodeling in various tissues. PAK1 may have other roles in GLUT4 translocation. The Ras family small GTPase RalA has been implicated in the regulation of GLUT4 vesicle tethering downstream of Rac1, but a Rac1 target that links Rac1 to RalA remains unknown.

Cytoskeletal remodeling is a crucial event that occurs when insulin stimulates glucose uptake in skeletal muscle and adipocytes (Figure 3) [34,35,36,37]. In particular, formation of microtubule and actin filament networks is important because GLUT4-containing vesicles are transported by kinesin (KIF3 and KIF5b) and myosin (Myo1c, Myo5a, and Myo5b) motor proteins that move along microtubule and actin filament tracks, respectively [38,39,40,41,42,43]. Furthermore, the actin cytoskeleton is required for retention of GLUT4 vesicles beneath the plasma membrane [44]. In many types of cells, cytoskeletal rearrangements are regulated by Rho family GTPases, and therefore, it is likely that these GTPases are also involved in the regulation of GLUT4 redistribution to the plasma membrane.

Roles of Rho family proteins in insulin-promoted glucose uptake seem to be cell-type specific. In adipocytes, TC10 has been implicated in a PI3K-independent pathway that regulates glucose uptake [45,46]. However, in skeletal muscle, TC10 is not involved, but instead, another Rho family member Rac1 has a pivotal role in insulin-dependent glucose uptake (Figure 3) [35,47,48,49,50,51]. The involvement of Rac1 in adipocyte insulin signaling was not supported by previous studies [52,53], but a recent study suggests a role of Rac1 in adipocytes [54]. P-Rex1, a Rac1-specific GEF, enhanced insulin-induced GLUT4 translocation downstream of PI3K in a Rac1-dependent manner in adipocytes [54]. Knockdown of P-Rex1, on the other hand, suppressed insulin-induced glucose uptake [54]. In addition, the *P-Rex1* gene has been mapped to a type 2 diabetes susceptibility locus [55]. These results support the notion that P-Rex1 and its substrate Rac1 are involved in insulin-dependent GLUT4 translocation. Hence, the involvement of Rac1 in adipocyte insulin signaling remains controversial.

Regulation of cytoskeletal rearrangements is an important role of TC10 and Rac1. However, both GTPases are also reported to regulate other processes prerequisite for redistribution of GLUT4 (see below). Thus, it is important to further clarify the detailed mechanisms how these GTPases exert their multiple functions through diverse target proteins.

## 6. Rac1 as a Critical Regulator for Insulin-Dependent Glucose Uptake in Skeletal Muscle

An array of studies using myocyte cultures suggest the involvement of Rac1 in skeletal muscle insulin signaling. Insulin caused Rac1 activation [47,50], and ectopic expression of a constitutively activated Rac1 mutant induced GLUT4 translocation [50,56]. Additionally, blockade of Rac1 function by siRNA-based knockdown or overexpression of a dominant-negative mutant remarkably suppressed insulin-stimulated GLUT4 translocation [47,48,49,50].

Evidence for a role of Rac1 has been provided also by studies using mouse and human mature skeletal muscle. The activation of Rac1 after intravenous injection of insulin was detected in mouse skeletal muscle [51]. Incubation of isolated skeletal muscle with insulin also induced Rac1 activation [57]. Furthermore, a hyperinsulinemic euglycemic clamp caused the activation of Rac1 in human vastus lateralis muscle [57]. On the other hand, GLUT4 translocation to the sarcolemma in mouse skeletal muscle was evaluated by immunofluorescence microscopy using an exofacial epitope-tagged GLUT4 reporter [51,58]. A constitutively activated mutant of Rac1, when ectopically expressed in mouse gastrocnemius muscle, induced GLUT4 translocation to the sarcolemma [51]. Moreover, insulin-stimulated GLUT4 translocation was markedly inhibited in *rac1* knockout mouse gastrocnemius muscle [51]. Similar results were obtained from immunogold electron microscopic analysis of endogenous GLUT4 [51]. Glucose uptake was also measured in soleus and extensor digitorum longus muscle isolated from wild-type and muscle-specific *rac1* knockout mice [57]. In fact, Rac1 deficiency caused a decrease in insulin-stimulated glucose uptake [57]. Chemical inhibitors for Rac1 also reduced insulin-dependent glucose uptake in isolated skeletal muscle [57]. These results altogether are supportive of a role for Rac1 in insulin-dependent glucose uptake in skeletal muscle.

Whole body glucose metabolism may be affected by *rac1* knockout if Rac1 really has an important role in glucose uptake. As expected, muscle-specific *rac1* knockout mice was reported to exhibit high plasma insulin concentrations after glucose injection and decreased glucose tolerance [57]. In addition, the activation level of the protein kinase PAK1, which is known as a direct target of Rac1, was lower in insulin resistant human and mouse skeletal muscle [57].

## 7. Mechanisms for Rac1 Activation in Response to Insulin Stimulation

The involvement of PI3K in the activation of Rac1 following insulin stimulation has been demonstrated by studies using specific inhibitors [47,50]. In addition, it has been shown that a constitutively activated mutant of PI3K induces Rac1 activation [59,60,61]. Therefore, it is feasible that Rac1 is regulated downstream of PI3K in insulin signaling. However, the detailed mechanisms underlying PI3K-dependent activation of Rac1 remain unclear and controversial.

In one proposed model, Akt2 and Rac1 independently function in distinct pathways that are bifurcated downstream of PI3K [35,56,62] (Figure 2A). Akt2 is thought to enhance GLUT4 vesicle trafficking through phosphorylation of diverse targets including AS160. On the other hand, Rac1 is thought to participate primarily in the regulation of cytoskeletal rearrangements. It is also suggested that products of PI3K, such as phosphatidylinositol-3,4,5-trisphosphate and phosphatidylinositol-3,4-bisphosphate, are involved not only in the activation of Akt2, but also in Rac1 activation, although the GEF responsible for this regulation of Rac1 remains to be identified. The observation that a constitutively activated Rac1 mutant can induce glucose uptake, when ectopically expressed alone, is explained by insulin-independent activation of Akt2 caused by PI3K that is stimulated through an unknown mechanism triggered by Rac1 superactivation [56]. Chemical inhibitors for Akt2 and Rac1 additively reduced glucose uptake in mouse soleus and extensor digitorum longus muscles following insulin stimulation, suggesting independent actions of Akt2 and Rac1 [62].

In another proposed model, Akt2 and Rac1 function in tandem, constituting a signaling cascade together with intermediary elements [59,60,61] (Figure 2B). Rac1 activation by insulin stimulation or ectopic expression of constitutively activated PI3K was blocked by knockdown of Akt2 [59,60]. Furthermore, GLUT4 translocation induced by constitutively activated Akt2 was totally down-regulated when Rac1 was knocked down in cultured myoblasts and mouse gastrocnemius muscle [60]. Collectively, these observations suggest a crucial role for Akt2 in insulin-stimulated Rac1 activation. In addition, phosphorylation of the activation segment of Akt2 was observed after intravenous insulin administration in not only wild-type, but also *rac1* knockout, mouse gastrocnemius muscle, consistent with the notion that Rac1 acts downstream of Akt2 [51].

In the latter (“tandem”) model, the Dbl family GEF FLJ00068 (also termed PLEKHG4 and puratrophin-1) has been implicated as a GEF responsible for the regulation of Rac1 downstream of Akt2 in insulin signaling in cultured myocytes [50]. FLJ00068 is expressed in a variety of tissues including skeletal muscle, and its Dbl homology domain exhibits substrate specificity toward Rac1 and Cdc42 [50,63]. Knockdown of this GEF by RNA interference caused significant reduction in the level of insulin-dependent GLUT4 translocation [50]. Enhancement of insulin-stimulated Rac1 activation and GLUT4 translocation was indeed observed when FLJ00068 was over-expressed [50]. Furthermore, a constitutively activated mutant of FLJ00068 induced Rac1 activation and GLUT4 translocation [50]. Therefore, FLJ00068 is a feasible candidate for the regulator of Rac1 in skeletal muscle insulin signaling. In support of this idea, constitutively activated FLJ00068-dependent GLUT4 translocation to the sarcolemma was in fact observed in wild-type, but not muscle-specific *rac1* knockout, mouse skeletal muscle [64]. Tiam1 is also reported to act as a GEF for Rac1, leading to GLUT4 translocation in muscle cells [56].

Although the evidence for the involvement of FLJ00068 in the regulation of Rac1 in skeletal muscle insulin signaling has been provided, the mechanisms underlying Akt2-dependent activation of FLJ00068 remain obscure. Dbl homology/pleckstrin homology domains exist in the *C*-terminal region of FLJ00068, and deletion of the *N*-terminal region renders this GEF constitutively active [50]. Therefore, it is reasonable to think that the *N*-terminal region negatively regulates GEF activity, which is increased through conformational change of the protein. FLJ00068 may not be phosphorylated by active Akt2 directly because the consensus sequence for Akt substrates is not found in FLJ00068.

## 8. Signaling Pathways Downstream of Rac1 for the Induction of Glucose Uptake

One important function of Rac1 in glucose uptake in muscle cells is the regulation of actin cytoskeletal rearrangements. Actin filaments serve as a track along which GLUT4 vesicles are transported. In muscle cells, the myosin motor Myo5b is responsible for GLUT4 vesicle trafficking along cortical actin filaments [40,65]. Moreover, the retention of GLUT4 vesicles beneath the plasma membrane and subsequent fusion steps require cortical actin remodeling, and Rac1 is indeed involved in these processes [44]. The motor protein Myo1c may link GLUT4 vesicles to actin filaments in the tethering step [38,65,66]. The involvement of Rac1 in actin cytoskeletal rearrangements prerequisite for the induction of glucose uptake is also supported by a pharmacological study in mature skeletal muscle [62].

Considering that ectopic expression of a constitutively activated mutant of Rac1 causes GLUT4 translocation in cultured myocytes and mouse skeletal muscle [50,51,56], Rac1 may direct not only cytoskeletal rearrangements, but also other processes required for GLUT4 translocation. The Ras family small GTPase RalA may play a key role downstream of Rac1, participating in the regulation of cell responses other than cytoskeletal remodeling (Figure 3). In many types of cells, RalA is localized in exocytic vesicles, and regulates polarized membrane transport and secretion, including synaptic vesicle transport in neuronal cells and the basolateral delivery of membrane components in epithelial cells [67]. RalA also controls receptor-mediated endocytosis [67]. A critical role of RalA in GLUT4 vesicle trafficking was first reported in adipocytes [66,68]. RalA is present in GLUT4 vesicles, and is activated upon insulin treatment. Activated RalA in turn associates with Sec5 and Exo84 subunits of the exocyst complex, and tethers GLUT4 vesicles to the plasma membrane before subsequent fusion events [66]. Not only the GDP/GTP cycle of RalA, but also the protein kinase C-dependent phosphorylation/dephosphorylation cycle of its effector Sec5, affect the interaction of RalA with the exocyst complex [68]. The motor protein Myo1c, which is known to connect GLUT4 vesicles to the actin filament [38], also binds to RalA in a GDP/GTP status-independent manner. In this way, GLUT4 vesicle transport along actin filaments and its tethering to the plasma membrane are cooperatively regulated by RalA [66].

In myoblast cells, an important role of RalA downstream of Rac1 in the control of GLUT4 translocation is demonstrated [69]. Insulin stimulation and ectopic expression of constitutively activated Rac1 resulted in the accumulation of the GTP-bound form of RalA in the membrane ruffling area [69]. On the other hand, constitutively activated Rac1-induced GLUT4 translocation was abrogated by knockdown of endogenous RalA [69]. Thus, RalA may be implicated in Rac1-dependent GLUT4 vesicle tethering to the plasma membrane in muscle cells. However, detailed mechanisms whereby Rac1 regulates RalA activity remain to be clarified.

Among diverse targets of activated Rac1, only PAK1 (other than regulators of the cytoskeleton) has been suggested to be responsible for the regulation of glucose uptake (Figure 3) [70,71]. *Pak1* knockout mice indeed showed peripheral insulin resistance coupled to this defect [71]. In addition, PAK1 activation in response to insulin was lowered in humans in acute and chronic insulin resistant states [57]. PAK1 is also reported to participate in AMP-activated protein kinase (AMPK)-mediated glucose uptake in muscle cells [72]. Taken together, PAK1 may play a pivotal role in glucose uptake as a target of activated Rac1. However, it is reasonable to think that PAK1 activation may not be sufficient for the induction of glucose uptake, considering a previous report that a constitutively activated mutant of Cdc42, another activator of PAK1, cannot induce GLUT4 translocation [50].

## 9. Role of Rac1 in Glucose Uptake Stimulated by Muscle Contraction

It is well known that not only insulin stimulation, but also contraction, cause glucose uptake in skeletal muscle. However, the mechanism underlying contraction-dependent glucose uptake seems to be different from insulin signaling. A signaling pathway specific to contraction-triggered responses involves AMPK, which acts as a cellular energy sensor. AMPK is activated in response to an increase in the intracellular AMP:ATP ratio, and further activation is achieved through phosphorylation by the serine/threonine kinase LKB1 [73,74,75].

A recent study demonstrates a critical role of Rac1 in contraction-dependent glucose uptake in skeletal muscle based on the analysis of muscle-specific *rac1* knockout mice [76]. Contraction-stimulated glucose uptake was blocked in skeletal muscle of *rac1* knockout mice, and exercise indeed caused the activation of Rac1 in both mice and humans [76]. AMPK is reported not to be involved in contraction-dependent Rac1 activation, and the precise mechanism whereby contraction leads to Rac1 activation remains unclear [76].

The biguanide metformin is known to induce glucose uptake via AMPK activation in muscle cells. Recently, an important role of the Rac1-specific GEF Tiam-1 in metformin/AMPK-stimulated glucose uptake in muscle cells has been reported [72]. Hence, AMPK may also employ the Rac1 signaling cascade to promote glucose uptake.

## 10. The Akt2-Independent Pathway Involving the Rho Family GTPase TC10 in Adipocytes

In adipocytes, PI3K-dependent and -independent signaling pathways are triggered by insulin receptor engagement [46]. The PI3K-dependent pathway is common to skeletal muscle, containing PDK1 and Akt2 downstream of PI3K, whereas the PI3K-independent pathway is specific to adipocytes, involving the Rho family GTPase TC10 [45,46]. Both of these two pathways are implicated in the regulation of GLUT4 translocation to the plasma membrane. TC10 is activated following insulin stimulation through the formation of a signaling complex composed of the Cbl proto-oncogene product, CAP and CrkII adaptor proteins, and the GEF C3G [45]. This signaling complex is co-localized with TC10 specifically in caveolin-enriched lipid raft microdomains, accounting for the specific role of the TC10 pathway in fully differentiated adipocytes.

Activated TC10 regulates actin cytoskeletal remodeling. In particular, it has been shown that cortical and perinuclear actin polymerization are differentially regulated by TC10 [77]. Furthermore, TC10 enhances GLUT4 vesicle trafficking and fusion to the plasma membrane by two mechanisms. Activated TC10 specifically binds to the exocyst component Exo70, thereby recruiting the exocyst complex beneath the plasma membrane. GLUT4 vesicles are then tethered to appropriate sites of the plasma membrane through the interaction with the exocyst complex prior to the fusion step [46,78]. Another mechanism whereby TC10 stimulates GLUT4 translocation depends on the Rab5 family member Rab31 [79]. In unstimulated cells, Rab31 is activated by the GEF Gapex-5, thereby causing the retention of GLUT4 in the endosomal compartments. Following insulin stimulation, the active form of TC10 forms a complex with Gapex-5 and its binding partner CIP4, and sequesters Gapex-5 away from its substrate Rab31. Consequently, Gapex-5 fails to activate Rab31, causing the accumulation of GLUT4 in GSVs.

## 11. Conclusions

Recent studies have revealed that multiple small GTPases are involved in a diverse array of signal transduction pathways for glucose uptake in insulin-responsive tissues as described in this article. However, the mechanisms underlying the regulation of these small GTPases are only partly understood. In some cases, specific regulators, such as GEFs and GAPs, are identified, although the precise mechanism remains controversial. Future studies will provide a clue to the function and regulatory mechanisms for the signaling network involving small GTPases.
